# Excavation of *Pid3* Orthologs with Differential Resistance Spectra to *Magnaporthe oryzae* in Rice Resource

**DOI:** 10.1371/journal.pone.0093275

**Published:** 2014-03-28

**Authors:** Xiao Xu, Qiming Lv, Junjun Shang, Zhiqian Pang, Zhuangzhi Zhou, Jing Wang, Guanghuai Jiang, Yong Tao, Qian Xu, Xiaobing Li, Xianfeng Zhao, Shigui Li, Jichen Xu, Lihuang Zhu

**Affiliations:** 1 National Engineering Laboratory for Tree Breeding, Beijing Forestry University, Beijing, China; 2 State Key Laboratory of Plant Genomics, National Centre for Plant Gene Research, Institute of Genetics and Developmental Biology, Chinese Academy of Science, Bejing, China; 3 Rice Research Institute, Sichuan Agricultural University, Chengdu, China; 4 National Key Laboratory of Hybrid Rice, Changsha, Hunan, China; 5 Key Laboratory of Molecular Biology and Gene Engineering, College of Life Science, Nanchang University, Nanchang, China; 6 Beijing Institute of Genomics, Chinese Academy of Sciences, Beijing, China; Institute of Botany, Chinese Academy of Sciences, China

## Abstract

Twenty-six orthologs of the rice blast resistance gene *Pid3* from cultivated varieties and wild rice accessions distributed in different areas were cloned by allele mining. Sequence analysis showed that while each of the orthologous genes from *indica* varieties and most wild accessions encodes a complete NBS-LRR protein, each of the proteins encoded by those from *japonica* varieties and few wild rice accessions presents a premature termination. Eleven of the 26 orthologs were selected for blast resistance testing by transforming into the blast susceptible rice variety TP309, respectively. Inoculation of 23 *M. oryzae* strains collected from diverse regions of China to the respective transgenic plants revealed that 6 *Pid3* orthologs showed susceptible to all the tested strains, while the other 5 orthologs showed differential resistance spectra in a gradually spectrum-widen order as *Pid3-W3*, *Pid3-W4*, *Pid3-I3*, *Pid3-W5* and *Pid3-I1*. Amino acid sequences alignment of these orthologs indicated that the sequence diversities between the blast resistance orthologs were mostly located in the LRR domain such as the substitutions of Q694H,D856H,Q896R,D899E etc. However, the differences between the resistance orthologs and the susceptible ones were mostly located in the NBS domain. The present experiments provide an example of that the ortholog evaluation of plant *R* genes could be an efficient way to expand the rice blast resistance and some other plant disease resistance as well for breeding.

## Introduction

Rice blast, caused by the filamentous ascomycete *Mangnaporthe oryzae*, is the most devastating fungal diseases affecting rice production [1]. Up to now, at least 78 major blast resistance (*R*) genes have been identified and mapped genetically on 11 rice chromosomes in exception of chromosome 3. Among the mapped blast *R* genes, 24 have been cloned [2]–[5] and functionally analyzed. With these known and cloned blast *R* genes, rice blast resistance breeding has become much more effective than before by using molecule markers linked to the known blast *R* genes [6].

Most of rice blast resistance genes conduct their reactions against a specific part of *Mangnaporthe oryzae* strains, thus pyramiding different rice blast *R* genes would facilitate rice breeding towards more durable and broader resistance to rice blast. For example, the plants pyramided with *Pi1*, *Piz-5* and *Pi-ta* showed enhanced resistance as compared to those carrying one or two of them, and the combination of these three blast R genes could be then deployed into superior rice varieties by marker-aided selection (MAS) [7]. By crossing and backcrossing rice lines C101LAC, C101A51 and Jin 23B, which contain *Pi1, Pi2* and *Pi33* respectively, Chen *et al*. introgressed the three blast *R* genes into a receptor parent Jin 23B. The pyramided lines showed a wide blast resistance spectrum covering 96.7% of the tested blast strains, which was much higher than that of the initial single *R* gene lines respectively [8]. In 2010, Koide combined two major rice blast resistance genes, *Pish* and *Pib*, from two near isogenic lines, the resulted pyramided line expanded the resistance spectrum by 64.3% [9].

However, in the practice of resistance breeding, using a single *R* gene which has a broad resistance spectrum is more effective. Of the known rice blast *R* genes, *Pi1* [10], *Pi2* [11], *Pib* [12], *Pi9* [13], *Pi5* [14], *Pi-ta* [15], *Pik* [16] and *Pik-p* [17] have been characterised as *R* genes with a relatively broad blast resistance spectum, respectively. Though the most other blast *R* genes may have a relatively narrow resistance spectrum, several experiments have confirmed that their alleles or orhtologs showed varied blast resistance spectra. These allelic or ortholog genes would be born with abundant allelic variations in rice resources due to the co-evolution of rice and blast pathogen in different rice growing environments, and could be utilized as more efficient and important gene resources in the improvement of rice blast resistance [18]. For example, *Pi9*,*Pi2* and *Piz-t* are alleles from different rice resources while physically at the same gene locus on rice chromosome 6 with the sequence similarity up to 98.84%, but their resistance spectra are quite different, covering 93.7%, 92.2% and 54.5% of tested blast isolates, respectively [4], [19], [20]. The similar situation was also observed in *Pik, Pik-m, Pik-p*, *Pi1, Pi54* and *Pi54rh* [3], [5], [16], [17], [21], [22], which were all from the same chromosome locus but cloned independently from different varieties and showed differential resistance spectra [23].

*Pid3* (GenBank accession no.: FJ745364.1), was initially identified in an *indica* variety Digu by performing a genome-wide comparison of ‘9311’ (*indica*) and ‘Nipponbare’ (*japonica*) on the premise of the verification of obvious different resistance of *indica* and *japonica* varieties to *M. oryzae* strains collected from south and north China [24], and the *Pid3* functional orthologs were found widely present in most tested *indica* varieties and wild rice species [25]. Later, Lv *et al* [2] isolated an ortholog of *Pid3* from a common wild rice accession A4 (*O. rufipogon*) by sequencing-based allele mining, which confers a different resistance spectrum to a set of *M. oryzae* strains and was named *Pid3-A4*. The purpose here is to isolate more ortholog genes of *Pid3* from cultivated varieties and wild rice accessions, and to evaluate their respective blast resistance by gene transformation and blast inoculation. Based on the respective blast resistance spectra of the cloned orthologs, comparative analysis were conducted between the amino acid polymorphic sites of the identified NBS-LRR proteins and their respective blast strain-specific resistances.

## Materials and Methods

### Plant materials and rice blast strains

There are 10 cultivated rice varieties including 5 *indica* varieties and 5 *japonica* varieties used in this study, collected from south or north of China and other countries. The 10 common cultivated rice varieties are kept in our lab, which are used and planted in China widely. Seventeen wild rice accessions were presented by the professor Zhukuan Cheng's lab, Institute of Genetics and Developmental Biology and Lili Hao's lab, Beijing Institute of Genomics (Table S1 in File S1). The species were cultivated in an experimental field of the Institute of Genetics and Developmental Biology in Beijing under normal growing conditions.

Twenty-three *M. oryzae* isolates used in this study are listed in Table 1. Of them, 20 isolates were collected from south of China and other 3 isolates from north of China. Zhong-10-8-14 was the initial isolate for the determination of the *Pid3* gene [25].

**Table 1:** Rice blast resistance spectra of *Pid3* orthologs.

*M. oryzae* strains	Transgenetic lines											
	*Pid3-I1*	*Pid3-I3*	*Pid3-W3*	*Pid3-W4*	*Pid3-W5*	*Pid3-W8*	*Pid3-W9*	*Pid3-W10*	*Pid3-W14*	*Pid3-W16*	*Pid3-J1*	*J1*
Zhong-10-8-14	**R**	**R**	**R**	**R**	**R**	S	S	S	S	S	S	S
07-31-1-2	**R**	**R**	**R**	**mR**	**R**	S	S	S	S	S	S	S
03-10-76-3	**R**	**R**	S	**mR**	**R**	S	S	S	S	S	S	S
04-8-2-1	**mR**	S	S	**mR**	S	S	S	S	S	S	S	S
10-25-1-1	**R**	S	S	mS	**R**	S	S	S	S	S	S	S
10-32-2-1	**R**	S	S	S	**mR**	S	S	S	S	S	S	S
10-128-2-1	**R**	S	S	S	NA	S	S	S	S	S	S	S
10-62-3-1	**mR**	S	S	S	**mR**	S	S	S	S	S	S	S
10-117-17-1	S	S	S	S	S	S	S	S	S	S	S	S
03-11-37-1	**R**	S	S	mS	**R**	S	S	S	S	S	S	S
07-55-1-1	**R**	**R**	mS	**mR**	**R**	S	S	S	S	S	S	S
07-26-2-2	NA	**R**	**mR**	S	**R**	S	S	S	S	S	S	S
10-120-21-2	**R**	S	mS	NA	**R**	S	S	S	S	S	S	S
07-21-1-1	NA	**R**	**mR**	**R**	**R**	S	S	S	S	S	S	S
99-20-2	**R**	**R**	S	S	**R**	S	S	S	S	S	S	S
99-26-2	**R**	**mR**	**mR**	S	**mR**	S	S	S	S	S	S	S
03-10-66-1	**R**	**R**	S	S	**R**	S	S	S	S	S	S	S
Chuang ZB15	**R**	S	S	NA	S	S	S	S	S	S	S	S
97-27-2	**R**	**R**	**R**	**R**	**R**	S	S	S	S	S	S	S
Y34	**R**	**R**	mS	**R**	NA	S	S	S	S	S	S	S
B04	**R**	**R**	S	**R**	**R**	S	S	S	S	S	S	S
B16	**R**	**R**	S	**R**	**R**	S	S	S	S	S	S	S
B23	**R**	**R**	S	**R**	**R**	S	S	S	S	S	S	S

(S:susceptible; mS:medium susceptible; R:resistant; mR:medium resistant; NA:no result).

### Gene cloning

DNA was extracted from the fresh leaves of 27 cultivated and wild rice varieties. Forward primer Pid3F: 5′ - TTTCTAGAAGTAACACCCAAGGATAGGATAG - 3′ and reverse primer Pid3R: 5′ - CTGTCGACGAACGACAAGTGCGACATGATTG - 3′ were designed and used to amplify the full coding sequence of *Pid3* based on the published dada [2]. An *Xba*I and a *Sal*I recognition site (underlined) with two protecting bases (TT and CT) were added to their 5′ ends, respectively. PCR amplification was carried out using the following profile: initial DNA denaturation, 95°C for 4 min; followed by 30 cycles of denaturation, 95°C for 30 s; annealing, 58°C for 30 s; extension, 72°C for 3 min; and final extension at 72°C for 5 min. The PCR products were purified and sequenced respectively.

### Rice transformation

No intron is present in the *Pid3* gene. Therefore, the cloned gene fragments above were inserted into the binary vector pZH01 [26] through the *Xba*I and *Sal*I cloning sites. After sequence verification, the final construct was introduced into *Agrobacterium tumefaciens* LBA4404. *Agrobacterium*-mediated transformation was performed using calli derived from mature embryos of susceptible rice variety TP309 according to Hiei *et al* [27].

Positive transgenic plants were detected through the amplification of the marker gene hygromycin (HYG) in vector using forward primer HYG-F: 5′ - TGCGCCCAAGCTGCATCAT - 3′ and reverse primer HYG-R: 5′ - TGAACTCACCGCGACGTCTGT - 3′. PCR amplification was carried out using the following profile: initial DNA denaturation, 95°C for 4 min; followed by 35 cycles of denaturation, 95°C for 30 s; annealing, 58°C for 30 s; extension, 72°C for 30 s; and final extension at 72°C for 5 min. To confirm the positive transformants precisely, one cleaved amplified polymorphic sequence (CAPS) marker was designed based on the nonsense mutation locus of *Pid3* in TP309 variety. A 658-bp fragment was amplified using the primer Pid3C (Pid3C-F: 5′ - TACTACTCATGGAAGCTAGTTCTC - 3′ and Pid3C-R: 5′ - ACGTCACAAATCATTCGCTC - 3′) and then digested with *Bam*HI. The digested PCR products was resolved on 2% agarose gels as one band in TP309 variety and three premature termination *Pid3* orthologs of *Pid3-W9, Pid3-W14*, and *Pid3-J1* transformants while two bands in the other positive transgenic plants.

### Expression analysis of *Pid3* orthologs

RNA were isolated from leaf sheath tissue with the TRIzol reagent (Invitrogen, Carlsbad, CA) and cDNA was synthesized from poly(A)^+^ RNA using a cDNA synthesis kit (Transgen, Beijing). Semi-quantitative reverse-transcription (RT)-PCR was performed with the specific primer pair Pid3C for 30 cycles of amplification. Transcription of the Actin gene was used to normalize the cDNA levels with the primer pair 5′ - AGCAACTGGGATGATATGGA - 3′ and 5′ -CAGGGCGATGTAGGAAAGC - 3′. Amplification of the Actin gene was conducted for 27 cycles. And the over-expression of *Pid3* gene was detected by the primer pair Pid3D (Pid3D-F: 5′- GAATGCAAATGTTTGGTTCG-3′ and reverse primer Pid3D-R: 5′-CGCCACATCATAATTCCTTG-3′). RT-PCR was initiated with one cycle at 95°C for 2 min followed by 30 or 27 cycles at 95°C for 30 s, 58°C for 30 s, and 72°C for 45 s, and the reaction was terminated with a final extension at 72°C for 5 min. The PCR products were resolved on 1% agarose gels.

### Fungal inoculation

Rice seedlings at the tillering stage were inoculated by injection of 0.1–0.2 ml of a 2.5×10^5^ conidia ml^−1^ spore suspension in the field. The disease reaction was evaluated seven days after inoculation with the susceptible transgenic line, *Pid3-J1*, as a control. Leaves lesion types were observed and scored as resistance (R), medium resistance (mR), medium susceptibility (mS), and susceptibility (S), respectively.

### Computational analysis of DNA and protein sequences

Sequences were aligned using DNAMAN (http://www.lynnon.com/), Clustal X version 2.0 [28], DNAStar (https://www.dnastar.com/products/lasergene.php) and manually edited using BioEdit version 7.0.1 [29]. Protein motif search was performed using the SMART program (http://smart.embl-heidelberg.de/) and the RCM program (http://144.92.198.58/main/main.php). MEGA 5 [30] was used to build phylogenetic tree of *Pid3* orthologs. Sliding-window analysis, polymorphism and neutral tests of different regions of the *Pid3* orthologs were conducted using DnaSP version 4.0 [31].

## Results

### Sequence characteristics of the *Pid3* orthologs

In addition to previously reported *Pid3* (hereafter renamed as *Pid3-I3*) from the *indica* variety Digu [25] and *Pid3-A4* (hereafter renamed as *Pid3-W5*) from the wild rice accession A4 (*O. rufipogon*) [2], a total of 28 *Pid3* orthologs were cloned, including 19 from 16 wild rice accessions (the additional three orthologs were from the wild rice accession W1, W11 and W13, respectively), 4 from *indica* and 5 from *japonica* varieties by allele mining. The details for the varieties are listed in Table S1 in File S1. They are distinguished in geographic distributions.

The open reading frame (ORF) analysis revealed that 26 *Pid3* orthologs each consist of 2775 nucleotides while the other two from W1-2 and W16 each consist of 2778 nucleotides. The pairwise alignment of all 30 *Pid3* orthologs showed high homologous with an average identity from 99.0% to 100% at the DNA level. Of note, the ortholog sequences from W12 and I2 are identical, and those from J3 and J5 and from W11-1, J2 and J1 are the same too. Therefore, we have actually obtained 26 different *Pid3* orthologs.

### Nucleotide polymorphism of *Pid3* orthologs

**Figure 1:**
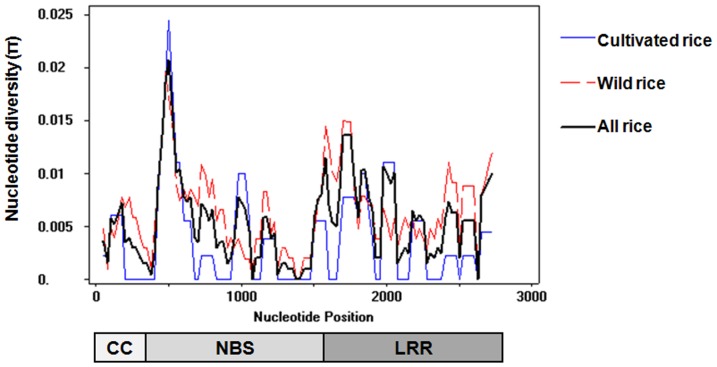
Sliding-window analysis of diversities in *Pid3* coding region. The nucleotide diversity (π; Y-axis) was generated by DNAsp5.0, and the X-axis represents the positions of nucleotides. The blue line stands for the result of cultivated rice varieties; the red line stands for the wild rice, and the black line stands for all rice. The map below the sliding window is the encoding structure of the *Pid3* gene, the shaded box denotes the exon region, and CC, NBS, LRR region are marked on its corresponding region.

*Pid3* genes encode a typical CC-NBS-LRR type protein with the CC, NBS and LRR domains coded in positions of 1-300, 481-1560 and 1621-2670, respectively, along the DNA sequence. By sliding window analysis of the 26 *Pid3* orthologs, the nucleotide diversity distribution in the whole encoding sequences was revealed in Figure 1, showing two obvious characteristics: firstly, a variation peak exists in the NBS domain; secondly, the nucleotide diversities of *Pid3* orthologs among the wild rice accessions are obviously greater than those among the cultivated rice varieties.

Further analysis was conducted in terms of different regions of *Pid3* orthologs by the neutral tests (Table 2). Tajima's D values were mostly negative but not significant, suggesting that *Pid3* orthologs may experience a little preference to positive selection but not balancing selection. The smaller π values (less than 0.5%) also showed the less variations for *Pid3* orthologs, while the nucleotide diversity in LRR domain of *Pid3* gene was larger than that in the CC and NBS domain. In *Pid3* orthologs of wild rice accessions, the non-synonymous substitution rate (Ks) of the LRR domain (−2.04091) and synonymous substitution rate (Ka) in the NBS domain (−2.10354) were statistically significant, but the K values in those of the cultivated rice varieties were not-significant.

**Table 2:** Polymorphism and neutral test of *Pid3* orthologs.

*Pid3* orthologs		Total sites	S	π	Tajima's D	Ka	Ks	Ka/Ks
All rice	Total	2778	110	0.585%	−1.63952	−1.58613	−1.63824	0.96819
	CC	300	12	0.473%	−1.76079	−1.84601	−0.88986	2.07449
	NBS	1080	37	0.481%	−1.71188	−2.06212*	−1.12712	1.82954
	LRR	1050	39	0.601%	−1.31948	−0.97568	−2.14213*	0.45547
Cultivated rice	Total	2778	27	0.372%	0.20057	0.04541	0.51546	0.08810
	CC	300	3	0.252%	−1.03446	−0.69098	−1.1173	0.62153
	NBS	1080	9	0.342%	0.69853	0.26384	0.93097	0.28341
	LRR	1050	10	0.351%	0.19314	0.19314	0	0
Wild rice	Total	2778	101	0.651%	−1.52933	−1.48135	−1.53105	0.96754
	CC	300	10	0.551%	−1.44664	−1.52506	−0.79238	1.92465
	NBS	1080	34	0.519%	−1.70834	−2.10354*	−1.11112	1.89318
	LRR	1050	37	0.666%	−1.30873	−0.99243	−2.04091*	0.48627

Note: S, number of segregating sites; π, nucleotide diversity; Statistical significance: * P<0.05; Not significant: 0.10>P>0.05; Ka: the rate of non-synonymous substitution, Ks: the rate of synonymous substitution.

### Amino acid sequence analysis of *Pid3* orthologs

Although the nucleotide sequences of *Pid3* orthologs showed highly homologous, their encoded amino acids display a distinct characteristics among *indica*, *japonica* varieties and wild rice accessions. The gene sequences from all the *indica* varieties and most of the wild rice accessions (15 of 19) encode the complete CC-NBS-LRR proteins with 924 amino acids but the gene sequences from all the *japonica* varieties and few wild rice accessions (4 of 19) showed premature transcription termination, which occurred at the position of 737 for individual wild rice accessions W11 and W17 and all *japonica* varieties, and at the position of 770 and 635 respectively for W14 and W9.

**Figure 2:**
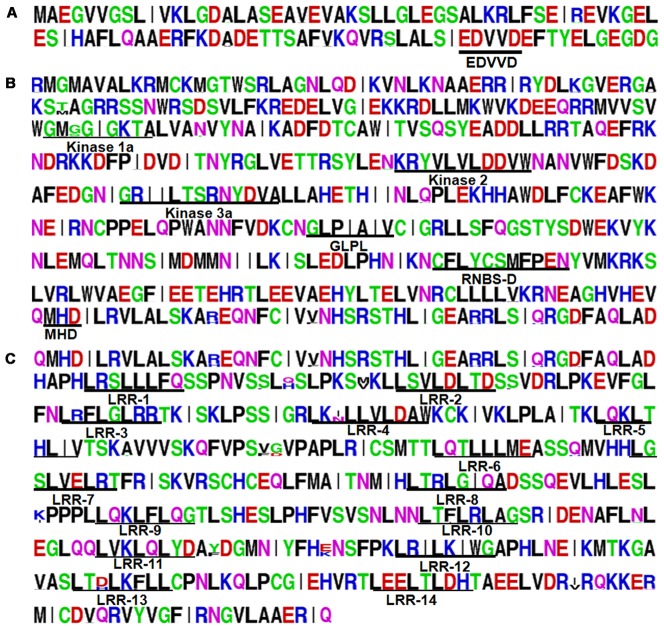
Conservatism analysis of amino acid sequences among *Pid3* orthologs. The conservatism analysis of amino acid sequences was produced by the software WebLogo on line. The piled height of amino acid loci showed the degree of conservatism, and the piled height of different amino acids in single locus reflected the degree of correlation with this site. (A): CC Domain. EDVVD was indicated in this domain; (B): NBS Domain. Several conserved motifs: Kinase 1a, Kinase 2, Kinase 3a, GLPL, RNBS-D and MHD were indicated in this domain; (C): LRR Domain. 14 imperfect LxxLxLxx repeat units were indicated in this domain.

Amino acid sequences alignment of Pid3 proteins revealed the homology rate, ranging from 97.9% to 100%. Several conserved motifs could be identified in the three functional domains, such as EDVVD in the CC domain (Figure 2A); Kinase 1a, Kinase 2, Kinase 3a, GLPL, RNBS-D and MHD in the NBS domain (Figure 2B); and 14 imperfect LxxLxLxx repeat units in the LRR domain (‘x’ indicates any amino acid residue) (Figure 2C). In the alignment, only few amino acid substitutions were observed in the motifs in addition to the positions of 204 (Kinase 1a), 311(Kinase 3a), 439 and 441 (RNBS-D) in the NBS domain, and the x loci of the third, fourth, eighth, tenth, thirteenth repeat unit in the LRR domain.

**Figure 3:**
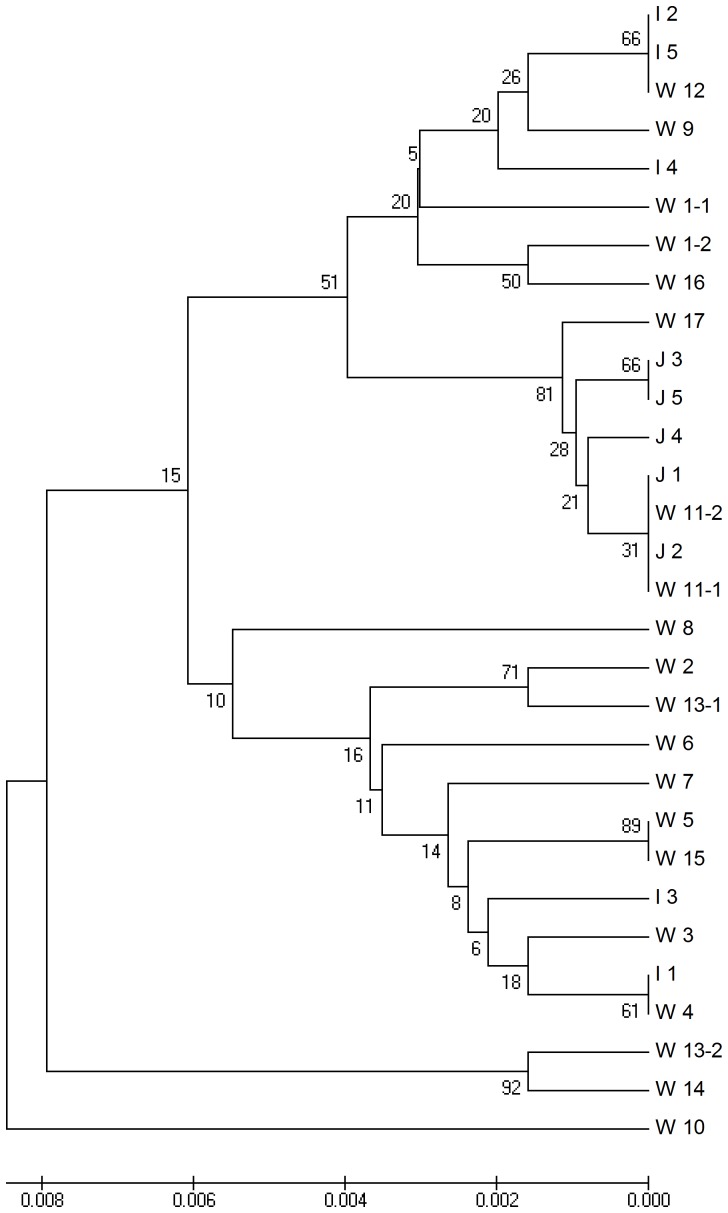
Phylogenetic analysis of Pid3 orthologs. W: wild rice; I: *indica*; J: *japonica.*

A neighbor-joining tree was constructed with the encoded amino acid sequences to evaluate the phylogenetic relationships of *Pid3* orthologs (Figure 3). *Indica*, *japonica* and wild rice varieties were relatively clustered in groups separately. The orthologs of most wild rice accessions were far from those of the cultivated varieties, but few ones were present between *indica* and *japonica* groups, which might reveal their possible genetic correlation in evolution.

### Blast resistance of *Pid3* orthologs

**Figure 4:**
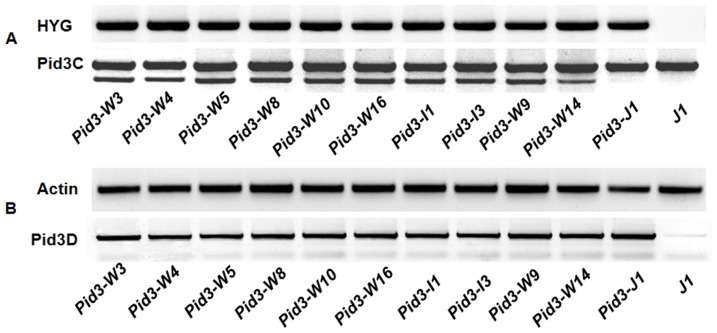
Detection of the DNA and RNA to the transformants. (A) HYG marker was used in the DNA detection. In addition, CAPS marker was used to confirm the positive transformants precisely and the restriction enzyme cutting site (*Bam*HI) lied in the premature stopped position of *Pid3-J1*; (B) Pid3D marker was used in the RNA detection, and Actin is as the referring primer marker.

**Figure 5:**
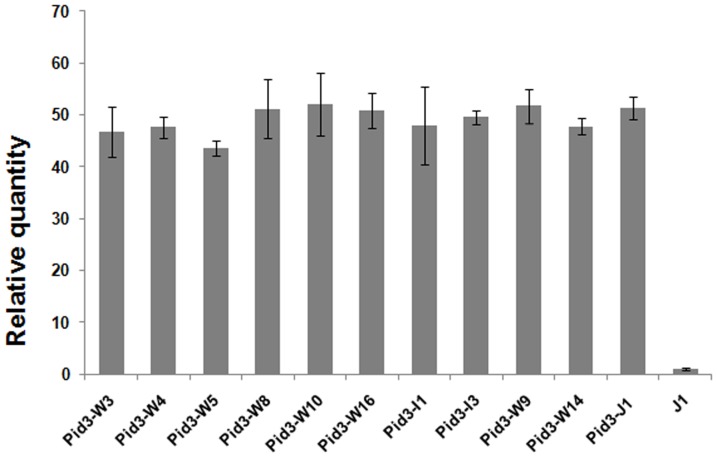
Quantitative reverse-transcription polymerase chain reaction analysis of the transcript levels of *Pid3* orthologs. Relative quantity charted the expression levels of *Pid3* orthologs in TP309 (J1). TP309 (J1) was used as the control.

Eleven *Pid3* orthologs, including 3 prematurely terminated *Pid3* orthologs from wild rice accessions of W9, W14 and *japonica* variety of J1 and 8 complete *Pid3* orthologs from wild rice accessions of W3, W4, W5, W8, W10, W16 and *indica* varieties I1 and I3, were selected for evaluation of their respective resistance to a group of rice blast strains based on their geographic distribution. To ensure the consistency, each of the *Pid3* orthologs were inserted into the binary vector pZH01 under Cauliflower mosaic virus (CAMV)35S promoter control and transformed into the susceptible rice variety TP309, which was the same recipient used for pCaMV35S::*Pid3* [25] and pCaMV35S::*Pid3-A4* [2] in our previous study. The independent primary transgenic lines (T0) were obtained and determined by the transgene CAPS marker (Figure 4A) and the transgene transcripts (Figure 4B). The results showed that all the candidate transformants were transgene-positive and over-expressed. The number of the positive transgenic plants in T0 generations was shown in Table S2 in File S1. Further, the transgenic lines, in which the respective transgenes were expressed at the approximately same levels (Figure 5), were selected for co-segregation analysis in their respective T1 (the selfed progeny of T0 line) generations (there was an example in Figure S1 in File S1) and then homozygous transgnic T2 (the selfed progeny of T1 line) lines were identified. The homozygous T2 lines from each of the 11 *Pid3* orthologs were applied to subsequent blast inoculation assays. As we expected, all of the *Pid3* orthologs transgenic plants did not present any observable side effects on phenotypes.

**Figure 6:**
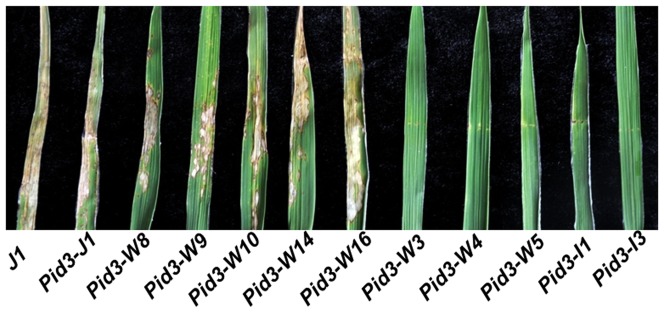
*Pid3* orthologs confer resistant and susceptible to the blast strain Zhong-10-8-14.

Next, all *Pid3* orthologs homozygous were inoculated with 23 *M. oryzae* strains respectively, which were collected from south and north of China. These blast inoculation experiments repeated 3 times on average and the results were listed in Table 1. Five completely encoded *Pid3* orthologs, *Pid3-W3, Pid3-W4, Pid3-W5, Pid3-I1* and *Pid3-I3*, were resistant to Zhong-10-8-14, the evaluating strain of the *Pid3* gene [2], while the other three completely encoded *Pid3* orthologs (*Pid3-W8, Pid3-W10* and *Pid3-W16*) and three premature terminated *Pid3* orthologs (*Pid3-W9, Pid3-W14* and *Pid3-J1*) were susceptible (Figure 6). In the further inoculation test, the 6 Zhong-10-8-14 susceptible *Pid3* orthologs also showed susceptibility to the other 22 *M. oryzae* strains, whereas the 5 Zhong-10-8-14 resistant *Pid3* orthologs displayed differential blast resistance spectra of the remaining strains: *Pid3-I1*, *Pid3-W5, Pid3-I3, Pid3-W4* and *Pid3-W3* were resistance to 19, 17, 13, 10 and 5 *M. oryzae* strains, respectively.

### Functional polymorphisms of *Pid3* orthologs

Of the 11 *Pid3* orthologs, Pid3-W9, Pid3-J1 and Pid3-W14 were premature terminated at the peptide position of 635, 737 and 770, respectively, resulting in deletions of 289, 187 and 154 amino acids, respectively, at the C-terminal of the Pid3 proteins, which may affect the function of the LRR domain.

Of the 8 completely encoded *Pid3* orthologs, 5 showed resistance to rice blast but with different spectra while the other 3 ones showed completely susceptible to all strains tested. In comparison of the protein sequences encoded by the two-group genes (resistance and susceptibility), the polymorphic loci were found mostly located in the NBS domain (Table 3), such as the G204C substitution in the Kinase1a motif of Pid3-W16 and the P441L substitution in the RNBS-D motif of Pid3-W10, indicating that the varied amino acids are important to the NBS domain's function.

**Table 3:** Comparison of Pid3 proteins.

Domain	CC				NBS													LRR																		
Amino	2	2	4	6	1	1	2	2	2	2	4	4	5	5	5	5	5	5	5	5	5	5	5	5	6	6	7	8	8	8	8	8	8	8	9	9
acid		2	4	6	5	9	0	0	5	9	2	4	1	1	3	3	3	5	5	6	7	7	7	8	2	9	9	1	2	5	5	9	9	9	0	0
position					3	1	3	4	9	7	3	1	1	8	2	6	7	1	3	9	1	3	7	9	5	4	9	5	4	1	6	4	6	9	0	6
Pid3-I1	-	V	R	A	T	E	M	G	I	D	L	P	L	N	I	R	R	H	P	S	H	L	V	S	N	Q	N	Y	E	V	D	I	Q	E	R	V
Pid3-W5	-	V	R	**V**	T	E	M	G	I	D	L	P	L	N	I	R	R	H	P	S	H	L	V	**L**	N	Q	**S**	**F**	E	**M**	D	**V**	Q	E	**Q**	V
Pid3-I3	-	V	**G**	A	T	E	M	G	**V**	D	L	P	L	N	I	R	R	H	P	S	H	L	V	S	N	Q	N	**F**	E	V	**H**	**V**	**R**	E	R	V
Pid3-W4	-	V	R	A	T	E	M	G	I	D	L	P	L	N	I	R	R	H	P	S	H	L	V	S	N	Q	N	Y	E	V	D	I	Q	**D**	R	V
Pid3-W3	-	**A**	R	A	T	**V**	M	G	I	D	L	P	L	N	I	R	R	H	P	S	H	L	V	S	N	**H**	N	**F**	E	V	**H**	**V**	Q	E	R	V
Pid3-W8	-	V	R	A	T	E	**V**	G	I	D	L	P	L	**S**	**T**	R	R	H	**L**	S	H	**S**	V	S	N	Q	N	**F**	E	V	**H**	**V**	Q	E	R	V
Pid3-W10	-	V	R	A	**M**	E	M	G	I	**E**	**F**	**L**	**P**	N	I	R	**C**	H	P	**L**	H	L	V	S	N	Q	N	**F**	E	V	**H**	**V**	Q	E	R	**A**
Pid3-W16	**A**	V	R	A	T	E	M	**C**	I	D	L	P	L	N	I	**H**	R	**Y**	P	S	**Q**	L	**M**	S	**I**	Q	N	Y	**K**	V	D	I	Q	E	R	V

Note: The bold markers were the diverse loci compared to Pid3-I1, which had the broadest resistant spectrum.

Among the 5 resistance *Pid3* orthologs with different resistance spectra, the polymorphic loci were mostly present in the LRR domain, implying that the region may function in recognizing the special blast strains. By comparison of the LRR domain sequences between the resistance orthologs, some amino acids could be recognized as important to the blast resistance spectra. For example, Q694H substitution that was only present in Pid3-W3, which showed the narrowest resistance spectrum; the D899E substitution between Pid3-I1 and Pid3-W4 led to a decrease of the resistance spectrum from 86.95% (*Pid3-I1*) to 47.82% (*Pid3-W4*); the substitutions, Q694H of Pid3-I3 and Q896R of Pid3-W3, altered their resistance spectrum from 60.86% (*Pid3-I3*) to 26.08% (*Pid3-W3*); and notably, the D856H substitution in either of Pid3-I3 and Pid3-W3, could result in a relatively narrow resistance spectrum.

## Discussion

### Allele mining is an important way to expand the rice blast resistance

**Figure 7:**
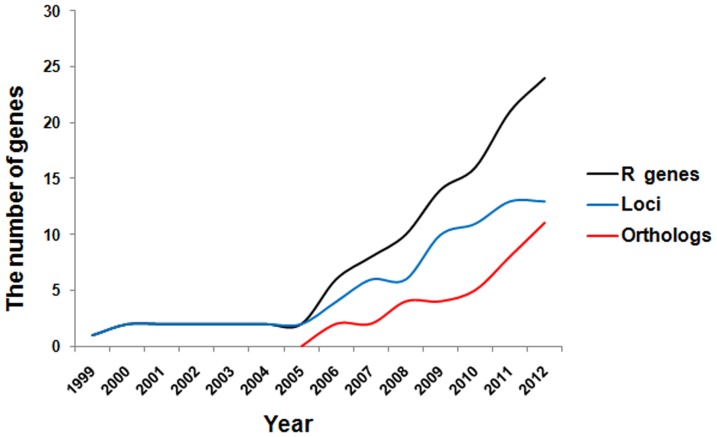
Cloned rice blast resistant genes and loci in proceeding. The black, blue and red line separately represents the total number of the *R* genes, *R* gene loci, and orthologs cloned year by year.

With the proceeding of the rice genome sequencing projects and the map-based cloning technology, over 20 rice blast resistance genes have been identified, but, in recent years, the number of new blast resistance loci have not increased as much (Figure 7). Some of the newly found genes were finally determined as an allele or an ortholog to one of the previously cloned genes [3]–[5], [32]. The blast resistance alleles or orthologs showing different resistance spectra from each other have thus greatly expanded the rice blast resistance resource for breading. This fact indicates that sequencing-based allele mining, as initially termed by Kumar *et al* [33], should be an efficient and economical way to expand the roles of each blast resistance loci in rice genome.

Some studies involving the silencing or over-expression of plant *R* genes have shown that disease resistance is correlated with *R*-gene expression, thus we have to admit that the acquisition of promoter sequences may play a crucial role to the functions of the involved *R*-genes in evolution. For example, a recent reported rice blast resistance gene, *Pit*, may be a result of the refunctionalization of some ‘sleeping’ rice blast *R* genes by transposon-mediated transcriptional activation in rice genome [34]. Here in current study, our research purpose was to evaluate and compare differential resistance spectra of *Pid3* ortholog sequences cloned directly by allele mining. Since the cloned ortholog sequences only involved their respective ortholog coding sequences, all the *Pid3* ortholog coding sequences were driven by the same CaMV35S promoter rather than their respective native promoters and were respectively expressed in transgenic rice plants of TP309 with much the same expression levels. This approach is an easy and effective way to explore possible available *Pid3* orthologs in spite of that some of the orthologs might be not functional in their own rice sources. Of course, we are unable to exclude the possibility that stronger expression of a *Pid3* ortholog in other rice plants might change its inherent blast resistance spectrum though such unusual situation did not occur in our previous studies [2], [25].

Thus, by this way, a total of 26 *Pid3* orthologs were cloned respectively from wild rice accessions and cultivated rice varieties distributed in different geographical areas, respectively. Eleven of them were tested for the rice blast resistance and showed different blast resistance spectra to these Chinese *M. oryzae* strains. The blast resistance spectra of *Pid3-I1* from Kasalath (*indica*) and *Pid3-W5* from *O. rufipogon* (A4) increased up to 86.95% and 78.26% respectively, as compared with the corresponding spectrum (60.86%) of *Pid3-I3*, the original *Pid3* gene from Digu. Noteworthily, *Pid3-I1*, which is from an Indian variety Kasalath, showed the best resistance to Chinese *M. oryzae* strains.

### Wild rice is an important resource for blast resistance

Sun *et al* [35], [36] reported that during rice domestication the number of alleles of cultivated rice was only 60% that of wild rice, leading to lower genetic diversity of the cultivated rice [37]. The two well-known rice *R* genes, *Xa21* for resistance to *Xanthomonas oryzae* and *Pi9* for resistance to *Mangnaporthe oryzae* were originated from the wild rice *O. longistaminat* [38] and *O. minuta* [39], respectively. They both showed broader-spectrum resistances to pathogens and have been widely applied to rice breeding. Thus the two *R* genes have set good examples for exploring more disease resistance genes in wild rice resources.

In plant, *R* genes were classified into four types according to their polymorphism level (π value): conserved (type I; π<0.5%), intermediate-diversified (type II; 0.5%<π<5%), highly diversified (type III; π>5%), and present/absent genes (type IV; P/A) [40], [41]. The higher π value means the evolution of the gene responding to the pathogen more rapidly. *R* locus with a low level of polymorphism usually has a relatively slow evolutionary rate. *Pid3* orthologs widely exist in the genomes of either cultivated rice subspecies or wild rice species. The present study showed that the π value, the nucleotide diversity, of *Pid3* orthologs in wild rice accessions is 0.651% (intermediate-diversified class) while 0.372% (conserved type) in cultivated rice varieties. Obviously, the diversity of wild rice is larger than that in cultivated rice, which supports that *Pid3* gene encoding sequences gradually tend to unity during the domestication of rice species. It also indicates that wild rice accessions are worthwhile for further allele mining of broad-spectrum *Pid3* gene orthologs.

In this study, we compared the blast resistance spectra of 5 *Pid3* orthologs, of which 3 were from wild rice accessions. As mentioned above, *Pid3-W5* had a blast resistance spectrum 17% wider than the original *Pid3-I3* from Digu. And *Pid3-W4* showed a resistance spectrum which is complement to that of *Pid3-I3*. Therefore, allelic mining from the wild rice is an effective way to expand rice blast resistance spectra in breeding.

### The amino acids variations in Pid3 protein domains relative to blast resistance and resistance spectra

In the neighbor-joining tree constructed with the amino acid sequences of all Pid3 proteins (Figure 3), the identified 5 resistant *Pid3* orthologs (*Pid3-W3, Pid3-W4, Pid3-W5, Pid3-I1, Pid3-I3*) are aligned in a branch, suggesting that some conservation in *Pid3* orthologs sequences is necessary for their resistance function. Based on such conservation, we may predict that in this group (Figure 3) the other two members, *Pid3-W7* and *Pid3-W15*, would have a blast resistance function too, though this prediction need to be further confirmed.

Many evidences showed that NBS domain in the resistance NBS-LRR proteins functions as a molecular switch in processing the resistance reaction, which decides the resistance level of the *R* genes [42]. In this study, of the 8 *Pid3* orthologs, each encoding a complete NBS-LRR protein, five were tested to be blast resistant while the other three were blast susceptible. When the susceptible protein sequences were compared with the resistance ones, the altered amino acids were found mainly in the NBS domain (as shown in Table 3, of total 13 substituted amino acids in the susceptible gene group, 11 were in NBS domain). Notably, only two substitution sites, G204 and, P441L are located in the conserved Kinase1a motif and RNBS-D motif, respectively, which suggest that amino acid substitution in other region of the NBS domain, besides the two conserved motifs, may also have the loss-of-function effects on the blast resistance of NBS-LRR proteins.

Many studies suggested that the LRR domain of *R* proteins is involved in avirulence (*AVR*) recognition or in physical interaction with *AVR* proteins, such as the recognition between barley *Mla1* and powdery mildew *AvrMla1* [43], potato *Rx* and *PVX* [44]; *Pita* and blast *AVR-Pita* [45]; flax *L* and rust *AVR-L567* [46]. However, in some cases, the CC domain was also found to play a major role in the interactions between *R* and *AVR* proteins. For example, in rice the *Pik* and *AVR-Pik* interaction involves the CC domain, but not the LRR domain, of the *R* protein [47]. Besides, Chen et al. [48] reported that the potato *RB* (also known as *Rpi-blb1*) gene encoded NBS-LRR protein binds to *Phytophthora infestans AVR* protein IPI-O through its CC domain.

In this study, we found that the amino acid variation in the LRR domain of the allelic Pid3 proteins greatly affects their resistance spectra. Within the 5 resistance *Pid3* orthologs, a total of 10 amino acid variations exist in the LRR domain, and accordantly their resistance spectra range from 86.95% to 26.08% of 23 tested blast isolations. Compared with the LRR domain of the original *Pid3* allele from Digu (*Pid3-I3*), which showed a resistance spectrum of 60.86% (14/23), two substitutions of Q694H and R896Q in *Pid3-W3* decreased the resistance spectrum to 26.08% (6/23); the substitutions of F815Y, H856D, V894I, R896Q, and E899D in *Pid3-W4* decreased the resistance spectrum to 47.82% (11/23). Meanwhile, some mutation sites increased the recognition to *M. oryzae* avirulence (*AVR*) elements and broadened the resistance spectrum, such as the substitutions of F815Y, H856D, V894I, and R896Q in *Pid3-I1* increased the resistance spectrum to 86.95% (20/23), the substitutions of S589L, N799S, V851M, H856D, R896Q, and R900Q in *Pid3-W5* increased the resistance spectrum to 78.26% (18/23). The data here may provide important clues for further exploring the core *AVR* recognition elements in the Pid3 proteins and understanding the interaction mechanism. Nevertheless, our study is preliminary, and the present data cannot exclude the possible role of the CC domain in the *AVR* recognition by Pid3 proteins.

Up to now, researches on the defense mechanism of rice blast caused by *Magnaporthe oryzae* have made great progress, resulting in identification and cloning of many defense related genes, which had been expected to facilitate the rice disease resistance breeding program via marker assisted selection and transgenic approaches [49]. Nevertheless, the actual progress in application of these defense related genes to breeding is rather limited. After evaluating the modification of the expression of more than 60 defense-related genes, Delteil et al [50] concluded that altered expression of genes involved in resistance signal transduction and transcription could lead to many unwanted side effects, like lesion mimic phenotypes, in contrast to *R* genes which could confer broad spectrum and high levels of resistance without obvious side effects. In this research, all *Pid3* orthologs transgenic plants though with the character of an over-expressed transgene did not present any observable side effects (data not shown). So far most of rice blast *R* genes have been cloned by map-based cloning approach, but this approach is labor-cost and time-consuming. The study here demonstrates that sequence-based allele mining (or coding sequence-based mining) is a more effective way to expand the rice blast *R* gene resource for controlling the devastating blast disease. This approach can be applied to control of other crop diseases too.

## Supporting Information

File S1 **Supporting information file containing Tables S1, S2 and Figure S1.** Table S1. Rice varieties and wild rice accessions used in this experiment. (DOC). Table S2. The number of the positive transgenic plants in T0 generations. (DOC). Figure S1. Co-segregation of the *Pid3-I1* gene with the resistant phenotype. The T1 generations (the selfed progeny of T0 line) carrying *Pid3-I1* were inoculated with *M. oryzae* Zhong-10-8-14, and the genotypes were analyzed using the HYG gene and marker Pid3C. Susceptible TP309 (J1) was used as the control. (TIF).
(RAR)
